# Acute Myocarditis-Like Episode in a Curly-Haired Young Boy—Red Flags for Familial Arrhythmogenic Cardiomyopathy

**DOI:** 10.3390/diagnostics10090651

**Published:** 2020-08-31

**Authors:** Alina Elena Pătru, Sebastian Onciul, Adrian Sturzu, Eliza Cinteză, Eleonora Gima, Bogdan A. Popescu, Philippe Chevalier, Ruxandra Jurcuț

**Affiliations:** 1 Expert Center for Genetic Cardiovascular Diseases, Emergency Institute for Cardiovascular Diseases, Sos. Fundeni nr. 258, 022328 Bucharest, Romania; alinucupopa@gmail.com (A.E.P.); sturzuadrian@ymail.com (A.S.); bogdan.a.popescu@gmail.com (B.A.P.); 2 Department 4-Cardiothoracic Pathology, University of Medicine and Pharmacy Carol Davila, Eroii Sanitari Bvd. 8, 050474 Bucharest, Romania; sebastian.onciul@gmail.com (S.O.); elizacinteza@yahoo.com (E.C.); 3 Department of Cardiology, Emergency Clinical Hospital Floreasca, Calea Floreasca nr 8, 014461 Bucharest, Romania; 4 Emerald Medical Center, Nicolae G. Caramfil, no 75, 077190 Bucharest, Romania; 5 Department of Pediatric Cardiology, Emergency Clinical Children’s Hospital “Marie S. Curie”, Bd. Constantin Brâncoveanu 20, 077120 Bucharest, Romania; 6 Department of Cardiology, University County Clinical Hospital, Bd. Tomis 145, 900591 Constanta, Romania; eleonora.gima@yahoo.com; 7 Department of Rhythmology, Hospices Civils de Lyon, Louis Pradel Cardiovascular Hospital, 26 Avenue du Doyen Jean Lépine, 69500 Lyon, France; philippe.chevalier@chu-lyon.fr; 8 Lyon Reference Center for Inherited Arrhythmias, Louis Pradel Cardiovascular Hospital, Université de Lyon, 28 avenue Doyen Lépine, 69500 Lyon, France

**Keywords:** arrhythmogenic cardiomyopathy, sudden cardiac death, myocarditis, cardiovascular magnetic resonance, cardiocutaneous syndrome, desmoplakin

## Abstract

The present case report describes a mother and son with arrhythmogenic cardiomyopathy (ACM) with early and greater left ventricle (LV) involvement. The presence of curly hair in both, together with the resuscitated sudden cardiac death of the mother, allowed timely genetic testing, which found a pathogenic nonsense mutation of the desmoplakin gene. While asymptomatic from an arrhythmic point of view, the son’s evolution was characterized by a well-documented exercise-induced myocarditis-like stage.

## 1. Introduction

Arrhythmogenic cardiomyopathy (ACM) is a primary heart muscle disorder, characterized pathologically by fibro-fatty replacement of the myocardium and clinically by a propensity towards ventricular arrhythmias, most often with a genetic cause [1]. Although the original disease phenotype was characterized by predominant right ventricular (RV) involvement, clinical variants characterized by early and greater left ventricle (LV) involvement, which may parallel (biventricular ACM) or exceed (left-dominant ACM) the severity of RV involvement, have been increasingly reported.

## 2. Case Reports

The family history started with the case of the patient′s mother, who, at age 38 (in June 2011) experienced a resuscitated cardiac arrest (ventricular fibrillation). The electrogradiographic (ECG) tracing after resuscitation showed sinus tachycardia with microvoltated QRS complexes in the precordial leads and newly diagnosed right bundle branch block morphology with a 2 mm ST segment elevation in the lateral leads. Transthoracic echocardiography (TTE) showed mild diffuse LV hypokinesia, with an estimated LV ejection fraction (EF) of 50% and a patchy hyperechogenic appearance of the interventricular septum (IVS). The RV dimensions and function were normal, and the RV free wall had a normal morphology and contractility. Coronary angiogram and 24 h ECG monitoring were unremarkable. Late ventricular potentials on signal averaged ECG were positive, being the only ACM minor criterion [2]. An internal cardiac defibrillator (ICD) was implanted for secondary prevention of sudden cardiac death (SCD).

**Figure 1:**
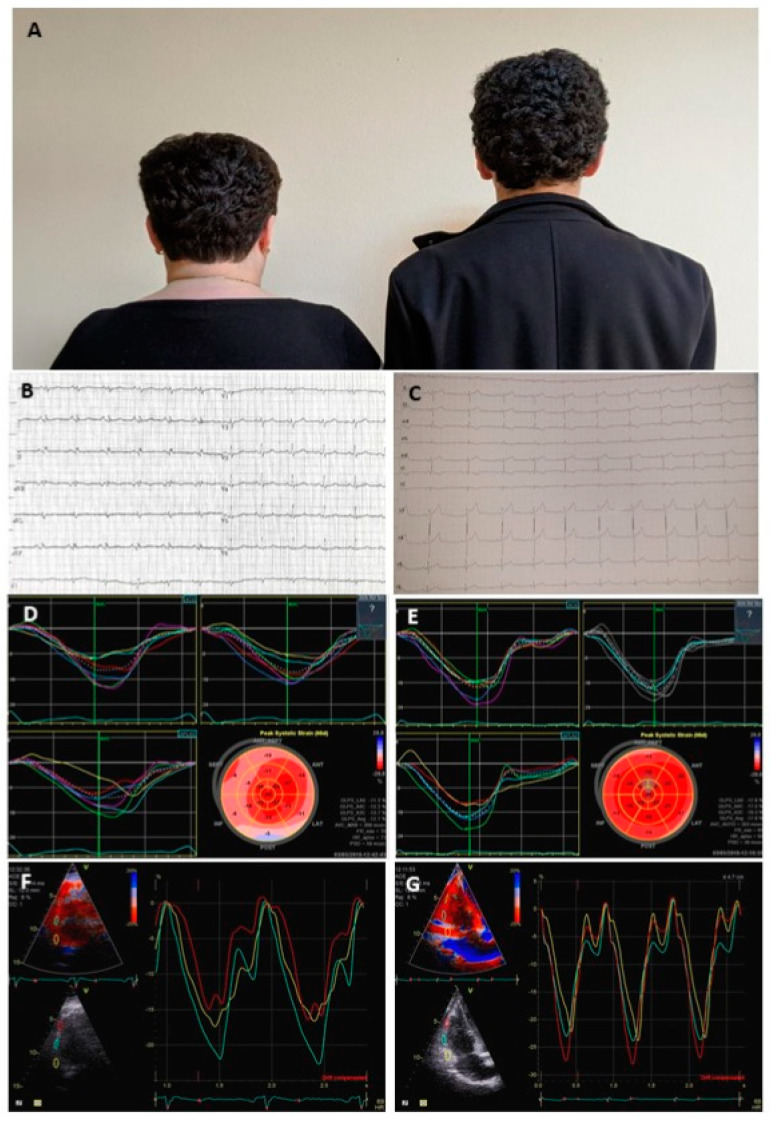
(**A**) Phenotypic findings in the mother and son: curly hair is noted; (**B**) electrocardiographic tracing of the mother: low voltage fragmented QRS complexes without ST-T changes. (**C**) Electrocardiographic tracing of the son: respiratory arrhythmia. (**D**) Speckle tracking echocardiography-derived left ventricle (LV) longitudinal strain segmental tracings and bull’s eye in the mother—showing abnormal myocardial amplitude in the basal and mid septal and lateral segments. (**E**) Speckle tracking echocardiography-derived LV longitudinal strain segmental tracings and bull’s eye with normal myocardial deformation in the son. (**F**) Free right ventricular (RV) wall tissue Doppler segmental strain tracings of the mother with abnormal amplitude of the deformation curves in the basal and apical segments. (**G**) Free RV wall tissue Doppler segmental strain tracings of the son with normal amplitude and shape of the curves. See text for further details.

In 2018, a patient and her 13 year-old son were referred to our center for a comprehensive evaluation. We noted their particular phenotype, both having curly hair since early childhood (Figure 1A), without any cutaneous particularities (no palmo-plantar keratoderma was present at examination in either mother or son). The three generations pedigree was not otherwise remarkable for cardiac disease (See Supplementary Figure S1).

At that time, the mother’s ECG showed low voltage fragmented QRS complexes without any ST-T changes (Figure 1B). Her TTE revealed a calculated LVEF of 53%, with basal IVS akinesia and hyperechogenicity and reduced LV global longitudinal strain (GLS) of −12.7% by speckle tracking echocardiography. It is more altered at the level of basal septal and lateral segments (Figure 1D; Supplementary Video S1) and the RV appeared mildly dilated with small free wall aneurysms and abnormal free wall longitudinal strain by tissue Doppler imaging (Figure 1F). Cardiac magnetic resonance (CMR) imaging could not be performed due to MR incompatibility of the ICD system. The 2010 Task Force criteria included one major criterion (dilated RV with free wall aneurysms) and one minor criterion (positive late ventricular potentials), suggesting a borderline diagnosis of ACM with biventricular involvement [2].

The son’s evaluation showed an unremarkable ECG (Figure 1C), while his TTE revealed indexed LV end diastolic volume (LVEDV) at the upper normal limit with preserved LVEF, LV GLS was −17.8% with lower than normal basal values (Figure 1E, Supplementary Video S2), and indexed RV dimensions at the upper normal limit without longitudinal strain abnormalities (Figure 1G).

Given the resuscitated SCD in a mother with borderline ACM criteria, as well as the curly hair of both family members, genetic testing was performed by next-generation sequencing (NGS) using a custom design based on a SeqCap EZ Solution-Based Enrichment strategy (Roche NimbleGen, Madison, WI, USA). The panel was designed to identify disease-causing mutations in 48 arrhythmia syndrome-causing genes (Supplementary Table S1). Target regions included coding exons (with a 30-bp padding) and 5′ and 3′ untranslated regions. Identified putative mutations were further verified using either Sanger sequencing for single-nucleotide variations and short indels, array comparative genomic hybridization methodology, or quantitative polymerase chain reaction for copy number variation. Among genomic variants identified for the proband, the only disease-causing mutation in the tested genes was a nonsense mutation of the desmoplakin (DSP) gene *p.Arg2284X* (c.6850C > T) on exon 24 in both mother and son.

**Figure 2:**
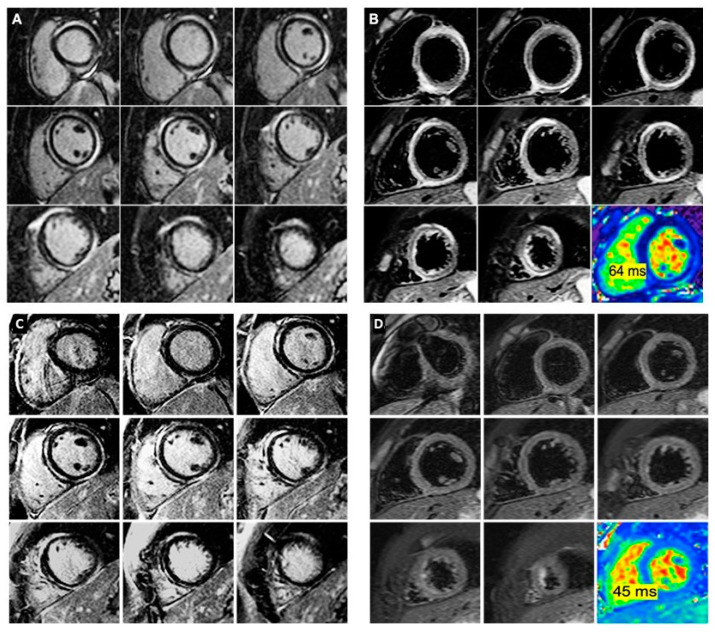
Contrast enhanced cardiac magnetic resonance during the chest pain episode (panels (**A**,**B**)) and at the 5-month follow-up (panels (**C**,**D**)). (**A**) Late gadolinium enhancement (LGE) imaging shows circumferential, subepicardial ring-like scarring of the left ventricle walls extending from the base to the apex. (**B**) Corresponding T2-weighted images showing areas of high T2 signal suggestive of myocardial oedema with similar topography to the scar. The corresponding short axis T2 map is displayed, showing increased T2 time of 64 ms. (**C**) Late gadolinium enhancement (LGE) imaging shows circumferential, subepicardial ring-like scarring of the left ventricle walls with the same extension as in the initial exam. (**D**) Corresponding T2-weighted images showing homogenous T2 signal, suggesting the absence of myocardial oedema. The corresponding short axis T2 map is displayed, showing normal T2 time of 45 ms.

In January 2020, after prolonged and sustained exercise (skiing), the son was admitted to the local pediatric service with chest pain and palpitations, with elevated cardiac biomarkers (troponin 4599 pg/mL, CK 1144 UI/mL, CK-MB 83 UI/mL), increased N-terminal pro-brain natriuretic peptide (NTproBNP) (841 pg/mL), and absent inflammatory markers (normal C-reactive protein, CRP, normal white blood cell count, WBC). His TTE showed preserved biventricular function. The CMR imaging showed a mildly dilated LV (LVEDV 94 mL/m²) [3] with mild systolic dysfunction (LVEF 54%) due to mild diffuse hypokinesia (Supplementary Video S3). The RV dimensions were normal (RV end-diastolic volume, RVEDV, 85 mL/m²), as well as its systolic function (RVEF 56%) (Supplementary Video S4). No regional dysfunction or structural alterations of the RV free wall were noted. However, late gadolinium enhancement (LGE) imaging showed an impressive amount of subepicardial, circumferential hyperenhancement with a “ring-like” appearance extending from the LV base to apex (Figure 2A). Oedema imaging showed areas of high T2 signal intensity overlapping the LV LGE hyperenhanced areas (Figure 2B). Of note, no areas of scar or oedema were noted in the RV free walls. Quantitative mapping identified markedly increased T1 and T2 values in the LGE hyperenhanced areas (1200 ms and 70 ms, respectively). His 24 h ECG monitoring showed less than 20 premature ventricular contractions (PVCs), with no episodes of ventricular tachycardia (VT). He was started on metoprolol 0.25 mg/kg/day and he was advised to avoid any strenuous exercise.

The 5-month follow-up CMR showed normal biventricular size and function, with the same pattern and extent of myocardial hyperenhancement on the LGE imaging (Figure 2C). However, there was complete remission of myocardial oedema on this second CMR, demonstrated by both the homogenous T2 signal on T2 weighted imaging and normal T2 mapping (45 ms) (Figure 2D).

## 3. Discussion

This family report illustrates the peculiar, incomplete, and polymorphic presentation of a severe form of ACM, with SCD as the first symptom in the mother, as well as acute myocardial inflammation and necrosis as the first presentation in the son. Moreover, it illustrates the use of advanced cardiac imaging, a particular pattern of progression of the left dominant ACM phenotype, characterized by intermittent exercise-induced myocarditis-like hot phases, followed by resolution of acute phenomena after exercise abstinence.

Two classical cardiocutaneous disorders with autosomal recessive inheritance, known as Naxos disease and Carvajal syndrome, are described, but additional cutaneous phenotypes can be linked to autosomal dominant DSP variants [1] and may present with SCD at early age, like the one illustrated by the presented family. A recent study described a 100% co-segregation of curly hair phenotype with DSP mutation carriers in ACM families, with palmoplantar keratoderma present to a lesser extent [4], similar to the present case report.

It is currently well established that ACM clinical course is characterized by intermittent myocarditis-like episodes, designated as “hot phases” outlined by troponin rise and myocardial inflammation [5,6]. These episodes may be recurrent, are frequently exercise-induced, and occur in the absence of any infectious triggers [5,7]. Conversely, genetic testing screening in six families, including patients with acute myocarditis associated with a family history of cardiomyopathy or SCD, revealed frequently arrhythmogenic variant carriers with left-dominant phenotypes, mainly with DSP variants [8]. While still considered rare in the ACM population, signs of acute myocarditis should draw the attention of the clinicians on this diagnostic possibility, especially when it occurs in relatives of patients with SCD, ventricular arrhythmia, or myocarditis history [9].

The frequent finding of lymphocyte infiltrates in myocardial pathology specimens of ACM patients does not necessarily indicate viral myocarditis and may be associated solely with myocyte death [10], which appears to be largely the result of apoptosis, especially in the early symptomatic phase of the disease. More recently, the DSP cardiomyopathy was characterized as a distinct form of ACM, characterized by episodic myocardial injury, left ventricular fibrosis that precedes systolic dysfunction, and a high incidence of ventricular arrhythmias [11]. Most of these findings are in contrast with those of the plakophilin-2 (PKP2) mutation carriers.

This case also illustrates the ability of CMR imaging to identify myocardial oedema in the acute phase of ACM, as well as its remission after exercise abstinence. We emphasize the impressive amount of LV scarring, resulting in only mild impairment of LV function and absence of acute heart failure symptoms, leading to the presumption of pre-existent fibrosis with additional recent superimposed necrosis and inflammation. The particular “ring-like” appearance of the subepicardial scar was strongly suggestive of a genetic ACM, specifically the previously diagnosed DSP mutation, as it was recently defined as an imaging hallmark for this genotype [12].

## 4. Conclusions

To conclude, prompt recognition of ACM red flags in affected patients and family members, followed by genetic testing, can be life-saving. Scenarios of recurrent myocarditis advocate for a thorough investigation of a possible ACM, as the disease clinical course appears to be characterized by myocarditis-like “hot phases” that may accelerate its progression.

## Supplementary Materials

The following are available online at https://www.mdpi.com/2075-4418/10/9/651/s1, Video S1: Transthoracic echocardiography of the mother—4 chamber view showing hypokinesia of basal septal and lateral segments; ICD lead visible in the right cavities. Video S2: Transthoracic echocardiography in the son—4 chamber view showing preserved LVEF RV dimensions at the upper normal limit without any contractility abnormalities. Video S3: Non-contrast cardiac magnetic resonance, cine images (balanced steady-state free precession, SSFP) in the 4 chamber view showing the normal size of both ventricles, with mildly impaired left ventricular (LV) systolic function (LV ejection fraction 54%) and normal right ventricle (RV) systolic function. Video S4: Non-contrast cardiac magnetic resonance, cine images (balanced SSFP) in right ventricle (RV) inflow-outflow view, showing no evidence of regional dysfunction or structural alterations of the RV walls. Table S1: List of arrhythmia syndrome causative genes tested Figure S1. Family pedigree for 4 generations with mother (III.2) and son (IV.1) marked as genotype positive—phenotype positive patients by the red filling.
