# Supplementary material for: Acute Myocarditis-Like Episode in a Curly-Haired Young Boy—Red Flags for Familial Arrhythmogenic Cardiomyopathy
Source: Diagnostics (Basel). 2020 Aug 31;10(9):651. doi: 10.3390/diagnostics10090651 (PMC7555819; doi:10.3390/diagnostics10090651)
Supplement: Supplementary file 1 [file diagnostics-10-00651-s001.zip › diagnostics-900961-supplementary table.docx]

**Supplementary Table S1.** List of arrhythmia syndrome causative genes tested

| **Gene** | **NM Number** |
| --- | --- |
| ***AKAP9*** | NM_005751.4 |
| ***ANK2*** | NM_001148.4 |
| ***CACNA1C*** | NM_001167625.1; NM_199460.3 |
| ***CACNA2D1*** | NM_000722.2 |
| ***CACNB2*** | NM_000724 |
| ***CALM1*** | NM_006888.4 |
| ***CALM2*** | NM_001743.4 |
| ***CALM3*** | NM_005184.2 |
| ***CASQ2*** | NM_001232.3 |
| ***CAV3*** | NM_001234.4 |
| ***CTNNA3*** | NM_013266.3 |
| ***DPP6*** | NM_130797.3 |
| ***DSC2*** | NM_024422.3 |
| ***DSG2*** | NM_001943.3 |
| ***DSP*** | NM_004415 |
| ***GJA1*** | NM_000165.4 |
| ***GJA5*** | NM_005266.6 |
| ***GPD1L*** | NM_015141.3 |
| ***HCN4*** | NM_005477.2 |
| ***JUP*** | NM_002230.2 |
| ***KCNA5*** | NM_002234.3 |
| ***KCND3*** | NM_004980.4 |
| ***KCNE1*** | NM_000219.5 |
| ***KCNE1L*** | NM_012282.2 |
| ***KCNE2*** | NM_172201.1 |
| ***KCNE3*** | NM_005472.4 |
| ***KCNH2*** | NM_000238.3 |
| ***KCNJ2*** | NM_000891.2 |
| ***KCNJ5*** | NM_000890.3 |
| ***KCNJ8*** | NM_004982.3 |
| ***KCNQ1*** | NM_000218.2 |
| ***RANGRF*** | NM_016492.4 |
| ***NOS1AP*** | NM_014697.2 |
| ***NPPA*** | NM_006172.3 |
| ***PKP2*** | NM_004572.3 |
| ***RYR2*** | NM_001035.2 |
| ***SCN10A*** | NM_006514.2 |
| ***SCN1B*** | NM_001037.4; NM_199037.3 |
| ***SCN2B*** | NM_004588.4 |
| ***SCN3B*** | NM_018400.3 |
| ***SCN4B*** | NM_174934.3 |
| ***SCN5A*** | NM_198056.2 |
| ***SLMAP*** | NM_007159.2 |
| ***SNTA1*** | NM_003098.2 |
| ***TGFB3*** | NM_003239.2 |
| ***TMEM43*** | NM_024334.2 |
| ***TRDN*** | NM_006073.3 |
| ***TRPM4*** | NM_017636.3 |
