# Supplementary figures and images for: Acute Myocarditis-Like Episode in a Curly-Haired Young Boy—Red Flags for Familial Arrhythmogenic Cardiomyopathy
Source: Diagnostics (Basel). 2020 Aug 31;10(9):651. doi: 10.3390/diagnostics10090651 (PMC7555819; doi:10.3390/diagnostics10090651)

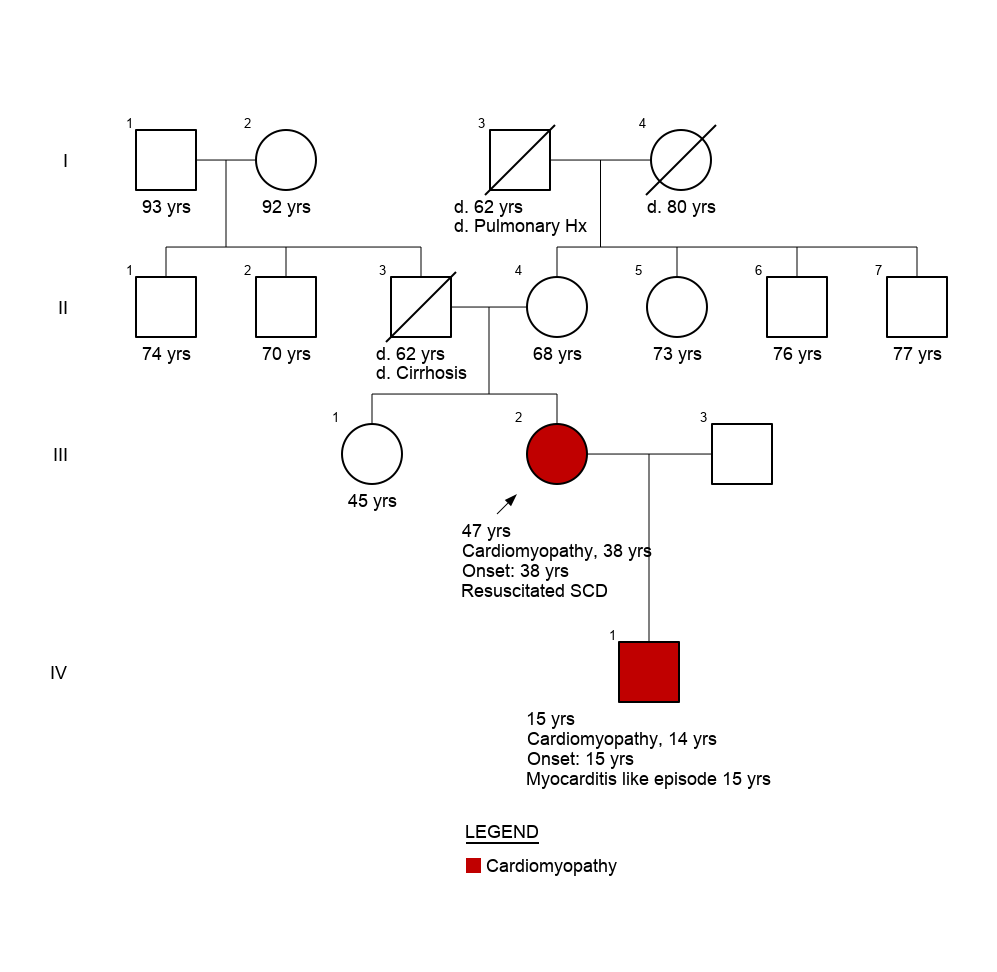

Supplement: Supplementary file 1 [file diagnostics-10-00651-s001.zip › diagnostics-900961-supplementary figure.jpg]
